# XPS Depth Profiling of Surface Restructuring Responsible for Hydrogen Evolution Reaction Activity of Nickel Sulfides in Alkaline Electrolyte

**DOI:** 10.3390/ma18030549

**Published:** 2025-01-25

**Authors:** Jiangtian Li, Deryn Chu, Connor Poland, Cooper Smith, Enoch A. Nagelli, Victor Jaffett

**Affiliations:** 1 U.S. Army DEVCOM Army Research Laboratory, 2800 Powder Mill Rd, Adelphi, MD 20783, USA; 2 Department of Chemistry & Life Science, United States Military Academy, West Point, NY 10996, USA; connor.poland@westpoint.edu (C.P.); cooper.smith@westpoint.edu (C.S.); enoch.nagelli@westpoint.edu (E.A.N.); victor.jaffett@westpoint.edu (V.J.)

**Keywords:** water splitting, hydrogen evolution, sulfides, surface restructuring, XPS depth profile

## Abstract

Electrochemical water splitting provides a sustainable method for hydrogen production. However, the primary challenge for electrochemical hydrogen generation is the high cost and limited availability of platinum-based noble-metal catalysts. Transition-metal chalcogenides have been identified as low-cost and efficient electrocatalysts to promote the hydrogen evolution reaction (HER) in alkaline electrolytes. Nonetheless, the identification of active sites and the underlying catalytic mechanism remain elusive. In this study, phosphorus-doped nickel sulfide has been successfully synthesized, demonstrating enhanced activity for alkaline HER. Investigating surface chemistry through X-ray photoelectron spectroscopy (XPS), depth profiling revealed that surface restructuring occurs during the HER process. The presence of phosphorus significantly influences this transformation, promoting the formation of a novel active Ni-O layer. This Ni-O layer is responsible for enhanced catalytic activity by upshifting the *d*-band center and increasing the density of states near the Fermi level, along with expanding the electrochemical surface area. This study reveals that the surface restructuring of transition-metal sulfides is highly tied to the electronic structure of the parent catalysts. Gaining a comprehensive understanding of this surface restructuring is essential for predicting and exploring more efficient non-precious transition-metal sulfide electrocatalysts.

## 1. Introduction

Hydrogen has emerged as a green energy carrier due to its high energy density and environmentally friendly properties. However, high-purity hydrogen generation, storage, and transportation have long been significant bottlenecks to the development and practical implementation of hydrogen fuel cell technologies [1,2,3,4,5,6,7,8,9,10]. Renewable electricity-powered water electrolysis to produce hydrogen is broadly recognized as a viable technology for a clean and sustainable global energy future [4,5,6,7,8,9,10], especially when considered to be integrated with renewable energy capture and onsite fuel cell systems. The key to enabling this technology lies in the development of low-cost and highly efficient electrocatalysts to accelerate water-splitting reactions. In particular, designing high-performance electrocatalysts for HER under alkaline conditions is more practical, as alkaline water electrolysis is the most commonly used approach in industry [10]. Nonetheless, alkaline HER suffers from more sluggish reaction kinetics compared to acidic electrolysis due to requiring an initial step to dissociate water molecules and thereafter create protons (H^+^) for subsequent H_2_ generation (H_2_O → H^+^ + OH^−^) [9,10,11,12,13,14,15,16,17]. To date, platinum-based precious materials are acknowledged as the benchmark electrocatalysts for HER thanks to their optimal adsorption/desorption energy with protons. Nonetheless, the widespread application of noble-metal-based electrocatalysts is dramatically hindered by their scarcity, leading to high costs. As a result, considerable efforts have been made to identify high-performance, earth-abundant, and cost-effective non-precious HER electrocatalysts [3,10,11,17].

In the last decade, transition-metal sulfides have been regarded as promising alternatives to platinum-group catalysts. Notably, nickel sulfides stand out for their excellent electrical conductivity, high redox current responses, and cost-effectiveness [18,19,20,21,22,23,24,25]. Ni_3_S_2_, a metal chalcogenide naturally occurring as the mineral heazlewoodite, exhibits intrinsic metallic behavior due to a continuous network of short Ni-Ni bonds throughout its structure [18]. In addition, Ni_3_S_2_ is the most nickel-rich phase in the stoichiometric formula, offering abundant Ni active sites [19,24]. Its high conductivity, coupled with its low cost, makes Ni_3_S_2_ well-suited for various electrochemical applications. While recently Ni_3_S_2_ based materials have been shown to be electrocatalytic active for either HER or OER, their catalytic activity and stability are still less competitive compared to those of the noble-metal catalysts.

Fundamentally, fostering catalytic activity in water splitting relies on optimizing the chemisorption of reactants and intermediates at the active sites. A variety of strategies have been employed to manipulate the electronic structures of nickel sulfides, aiming to regulate the adsorption behavior of intermediates on active sites and enhance HER performance. These strategies include the incorporation of foreign atoms, morphological engineering, and the construction of heterojunctions [18,19,20,21,22,23,24,25,26,27,28,29,30,31,32,33]. The formation of heterojunctions between Ni_3_S_2_ and either Ni or MoO_2_, for example, facilitated the adsorption and dissociation of water molecules into hydroxide ions (OH^−^) and protons (H^+^), thereby accelerating the hydrogen production process [20]. Additionally, the incorporation of the second cation (Fe, W, Sn, Mo, etc.) or ion (phosphorus, fluorine, and nitrogen) into sulfides has been revealed to enhance charge-transfer rates and increase the electrochemical surface area, resulting in improved HER activity [22,24,25,26,27,28,29,30,31]. Despite these significant efforts, the water-splitting performance of nickel sulfides remains inferior to that of noble-metal-based catalysts, and the identification of active sites and the underlying catalytic mechanisms remains elusive. These conclusions are traditionally drawn under the assumption that no surface changes occur during the electrochemical reactions. However, surface restructuring has been observed in several electrochemical reactions, such as the oxygen evolution reaction involving metal sulfides, phosphides, and nitrides, where the top surface is completely transformed into oxy-hydroxyl oxides as the active species, along with ion leaching [3,6,8,9,10,11,12,13]. Under oxygen reduction reaction (ORR) conditions, Ni_3_S_2_ has been found to undergo surface reconstruction, forming a highly active, amorphous NiS phase approximately 2 nm in thickness [8]. In another study focused on alkaline HER, NiS was shown to undergo an in situ phase transition, resulting in an intimately mixed phase of Ni_3_S_2_ and NiO. This phase transformation generates highly active synergistic dual sites at the Ni_3_S_2_/NiO interface [10]. Our recent work also observed surface reconstruction in 3D NiOS nanostructures for alkaline HER, leading to the formation of a Ni-O catalyst layer functionalized with low-coordinated oxygen atoms and abundant vacancies. These features enable active sites for H_ad_ adsorption/desorption, while nearby Ni^δ+^ vacancies facilitate optimal interactions with OH_ad_ [9]. These studies highlight the dynamic nature of transition-metal chalcogenides in electrocatalysis [8,9,10]. Despite the observed surface restructuring, several important questions remain unanswered, such as: What parameters govern the reconstruction? How can surface restructuring be controlled or predicted? And how can restructuring be directly linked to improved catalytic performance? Surface restructuring remains challenging to fully characterize and control, and a more comprehensive understanding is required. This is highly dependent on the clear observation and analysis of the chemical compositions and states at the surface. Among a variety of techniques employed for analysis, XPS is a highly sensitive surface analysis method. When combined with sputtering to gradually remove material between analysis cycles without damaging the underlying material, depth profiling allows for high-resolution analysis of the atomic composition and chemical state of the electrode with depth [14,15,16]. The information gained from this technique could significantly enhance our understanding of the compositional and structural changes that occur during surface restructuring, as well as provide insights into how to control this process.

Traditionally, catalyst electrodes are prepared by depositing catalyst ink, mixed catalyst powders with binders (commonly the Nafion), on glassy carbon. The co-existence of organic binders will interfere with the in/ex-situ characterizations and thereby obscure our understanding of the intrinsic activity and the potential surface reconstruction of the catalysts. In this study, instead, pure nickel sulfides were successfully fabricated by direct sulfurization of Ni foam under hydrothermal conditions and subsequently doped with phosphorus, which is able to create a clean surface and allows us to examine surface changes after HER without interference from conventional binders. We evaluated the electrocatalytic HER performance of these catalysts in an alkaline electrolyte, finding significant enhancements due to phosphorus doping. XPS depth profiling of the post-HER electrodes revealed that surface restructuring occurred during the alkaline HER process for both sulfide electrodes. Additionally, the presence of phosphorus significantly impacts this restructuring process on the top layer by introducing crystal defects through the doping effect while protecting the further proceeding to the deeper layer. This behavior contributes to the robust stability of sulfide catalysts.

## 2. Materials and Methods

### 2.1. Fabrication of Ni_3_S_2_ Framework

All chemicals in this study were purchased from Sigma-Aldrich (MilliporeSigma, St. Louis, MO, USA) and used as received. The fabrication of Ni_3_S_2_ is technically based on the sulfurization of Ni foam with a sulfur source. Typically, 3.41 g of thiourea was dissolved in 90 mL of DI water in a Teflon liner, and then a piece of pre-cleaned Ni foam with a geometric size of 6 × 3.5 cm^2^ was immersed in it. The Teflon liner was sealed in Autoclave and kept at 180 °C overnight. After it cooled down to room temperature, the reacted Ni foam was rinsed with DI water and ethanol and then dried under vacuum at 60 °C overnight.

### 2.2. Phosphorization of Ni_3_S_2_ (P-Ni_3_S_2_)

The phosphorization of Ni_3_S_2_ was carried out with NaH_2_PO_4_ as the phosphorous source. Typically, 1 g of NaH_2_PO_4_ in a ceramic boat was placed in a quartz tube, and one piece of Ni_3_S_2_ framework with a size of (3 × 3.5 cm^2^) was placed on the downstream side. The tube furnace was flushed with N_2_ for 30 min, then heated up to 300 °C with a ramping rate of 1 °C/min, and then kept for 2 h under N_2_ flow with a flow rate of 100 SCCM (Standard Cubic Centimeters per Minute). The sample was denoted as P-Ni_3_S_2_.

### 2.3. Characterizations

The crystal structure was acquired on a Rigaku X-ray diffractometer (Rigaku Flex 600, Rigaku, Tokyo, Japan) with Cu Kα radiation (λ = 1.54 Å) within the diffraction angle range of 20 to 80°. Morphology of samples was observed using a Zeiss Auriga 60 Field Emission Scanning Microscope (ZEISS, St. Louis, MO, USA), operating at an accelerating voltage of 10 keV. XPS measurements were conducted on a Physical Electronics VersaProbe III employing a monochromatic Al Kα source with an energy of 1486.6 eV. The analyzer pass energy was set to 224 eV for survey scans and 55 eV for high-resolution scans. All spectra were calibrated to the adventitious C 1s peak at 284.8 eV. For the XPS depth profile, all specimens were ion sputtered using an argon gun operating at 2 KeV over a 3 mm × 3 mm area for 5 cycles, with each cycle consisting of 4 min of sputtering and followed by data collection.

### 2.4. Electrochemical Performance

All electrochemical performance evaluations were carried out on a Gamry Reference 3000 (Gamry Instruments, Warminster, PA, USA) potentiostat. A three-electrode electrochemical cell was employed with the developed catalyst as the working electrode, the Ag/AgCl as the reference electrode, and the Pt wire as the counter electrode. 1 M KOH aqueous solution was used as the electrolyte during all electrochemical testing. Before conducting linear sweep voltammetry (LSV) with a scanning rate of 10 mV/s, chronopotentiometry was performed at −1.8 V vs. Ag/AgCl (−0.8 V vs. RHE) for 30 min to stabilize the current density. The potential vs. the Ag/AgCl electrode was calibrated to a reversible hydrogen electrode (RHE) with the following equation: E (RHE) = E (Ag/AgCl) + 0.059 pH + 0.197.

The electrochemical impedance spectroscopy (EIS) was conducted in a frequency range from 0.1 Hz to 100 kHz with an AC amplitude of 10 mV, and the electrocatalytic kinetics was studied by analyzing the semicircle of the Nyquist plot. The electrochemical double-layer capacitance (C_dl_) was determined by running cyclic voltammetry (CV) in the non-Faradaic region with different scan rates. The electrochemically active surface area (ECSA) was then calculated using the following equation: ECSA = C_dl_/C_s_, where the C_s_ is the constant specific capacitance of the smoothy electrode (60 µF commonly used).

### 2.5. Preparation of Catalyst Ink and Pt Electrode

As a control sample for comparison, a Pt electrode was also prepared. A total of 5 mg of commercial Pt (20 wt.%)/C catalyst was dispersed in a mixture of 500 µL ethanol and 500 µL DI water with 10 µL of 5 wt.% 117 Nafion solution. The mixture was sonicated for 30 min to yield a homogeneous catalyst ink. Thereafter, 10 µL of ink was dropped on glassy carbon with a diameter of 3 mm. That will act as the Pt working electrode.

## 3. Results and Discussion

### 3.1. Catalyst Structure

**Figure 1:**
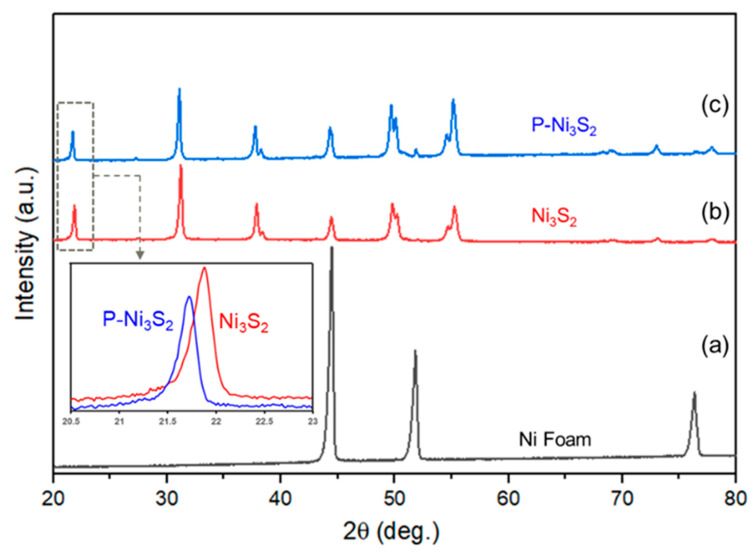
XRD patterns for Ni foam (**a**), Ni_3_S_2_ (**b**), and P-Ni_3_S_2_ (**c**). The inset compares the peak shift after P doping in Ni_3_S_2_.

**Figure 2:**
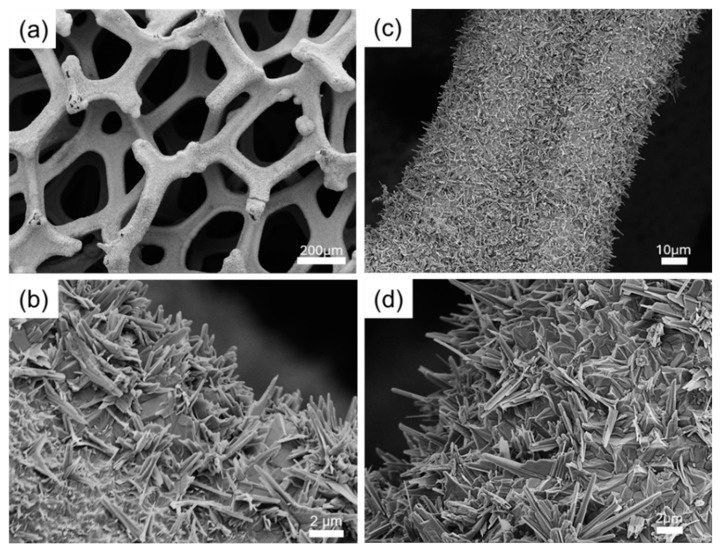
Surface morphology of developed catalysts. SEM images for freshly prepared Ni_3_S_2_ (**a**,**b**), and P-Ni_3_S_2_ (**c**,**d**), respectively, with different magnifications.

To investigate the evolution of phase structure, the obtained samples were first characterized by X-ray diffraction (XRD), as shown in Figure 1. The Ni foam displays three distinct diffraction peaks (Figure 1a) corresponding to the (111), (200), and (220) planes of metal nickel (JCPDS No. 04-0850) [20]. In the sulfide sample, all diffraction peaks are indexed to the crystalline phase of heazlewoodite Ni_3_S_2_ (JCPDS No. 44-1418) [19,20], with no detectable peaks from impurities (Figure 1b), indicating a complete sulfurization of the Ni foam to Ni_3_S_2_ under this reaction condition. As shown in Figure 1c, the doping of phosphorus into the Ni_3_S_2_ lattice does not significantly alter the crystal structure of the parent material. However, due to the larger atomic radius of phosphorus compared to sulfur, the incorporation of phosphorus expands the crystal structure, leading to increased *d*-spacing and a redshift of the diffraction peaks towards smaller angles, as highlighted in the inset of Figure 1. Notably, no nickel phosphides were formed during the phosphorization process, confirming successful phosphorus doping in Ni_3_S_2_. The evolution of morphology was examined using scanning electron microscopy (SEM). Figure 2a,b present SEM images at varying magnifications, illustrating that the Ni_3_S_2_ nanostructures effectively replicate the Ni foam skeleton without compromising its integrity during the sulfurization process. Notably, nanowires have grown on the surface, demonstrating excellent adhesion to the skeletal framework. This nanostructure offers distinct advantages over bulk materials, including a larger surface area and one-dimensional interwire channels, which facilitate electrolyte diffusion, enhance electron transport, and ensure ample contact between the active materials and the electrolyte [32]. The nanostructures were preserved in the phosphorus-doped catalyst P-Ni_3_S_2_, as shown in Figure 2c,d.

### 3.2. Surface Chemistry

**Figure 3:**
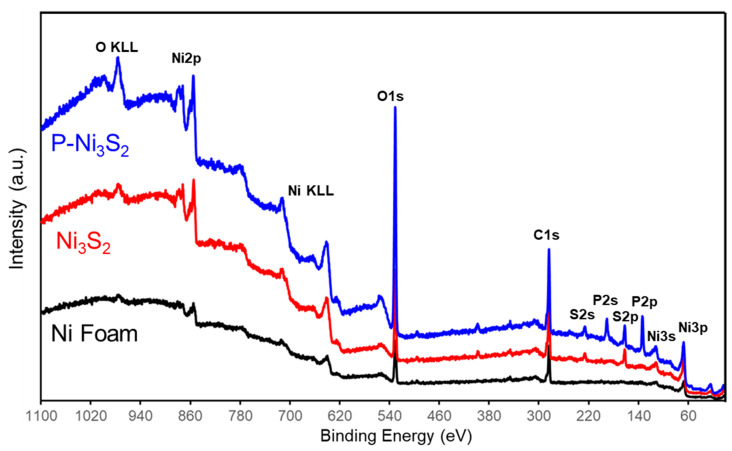
XPS surface survey of Ni foam, freshly prepared Ni_3_S_2_, and P-Ni_3_S_2_, respectively.

An X-ray photoelectron spectroscopy (XPS) survey was conducted to verify the chemical composition of all catalysts, as shown in Figure 3. The predominant contributions were observed from C 1s, O 1s, Ni 2p, and Ni LMM. In the Ni_3_S_2_ sample, distinct sulfur features, S 2s and S 2p are clearly visible, while the phosphorus features, P 2s and P 2p, appear after phosphorization within the binding energy range of 100 to 250 eV. This indicates the successful incorporation of phosphorus.

**Figure 4:**
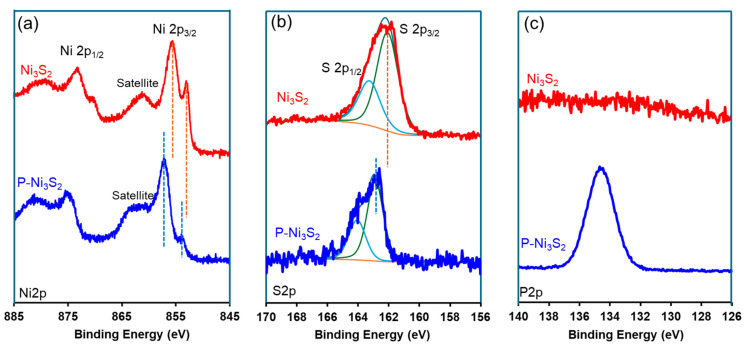
Surface chemistry of freshly prepared catalysts. High-resolution XPS spectra for Ni 2p (**a**), S 2p (**b**), and P 2p (**c**), respectively, in Ni_3_S_2_ (top) and P-Ni_3_S_2_ (bottom) prior to electrochemical testing.

To closely examine variations in chemical compositions, high-resolution XPS spectra were obtained and are shown in Figure 4 for Ni 2p (a), S 2p (b), and P 2p (c), respectively. The successful doping of phosphorus is confirmed by the emergence of a P 2p peak at 135 eV in the P-Ni_3_S_2_ sample, in contrast to the flat signal observed in the Ni_3_S_2_ sample (Figure 4c). As shown in Figure 4a, both Ni 2p XPS spectra exhibit two main bands in the ranges of 850–865 eV and 870–885 eV, corresponding to the Ni 2p_3/2_ and Ni 2p_1/2_ spin-orbit doublets, respectively, with a splitting of approximately 17 eV and a Ni 2p_3/2_ to Ni 2p_1/2_ ratio of 2:1 [23,26,33,34,35]. Focusing on the 2p_3/2_ region in the pristine Ni_3_S_2_ sample, peaks at 855.3 eV and 852.7 eV are characteristic of Ni_3_S_2_ and are assigned to Ni-S and Ni-Ni bonds, respectively [23,35,36]. The bump centered around 861 eV arises from the satellite peak of Ni 2p_3/2_. In the P-Ni_3_S_2_ sample, two major changes in the Ni 2p spectra are observed following phosphorus doping: (i) the Ni-S peak shifts to a higher binding energy by 1.6 eV, reaching 856.9 eV, and the Ni-Ni peak shifts by 1.1 eV to 853.8 eV; (ii) the Ni-Ni ratio significantly decreases. In the S XPS spectra (Figure 4b), the major band centered around 163 eV is attributed to the S 2p signal, which can be deconvoluted into two peaks: S 2p_3/2_ and S 2p_1/2_. In the P-Ni_3_S_2_ sample, the S 2p_3/2_ peak shows a positive shift to 162.8 eV, an increase of 0.7 eV compared to Ni_3_S_2_ due to the presence of phosphorus. Overall, the incorporation of additional phosphorus ions into the structure extracts more electrons from the Ni atoms. As a result, some Ni-Ni bonds are partially broken and reformed with phosphorus, leading to the delocalization of electrons among the Ni cations and sulfur ions [20,37].

### 3.3. Electrochemical Performances

**Figure 5:**
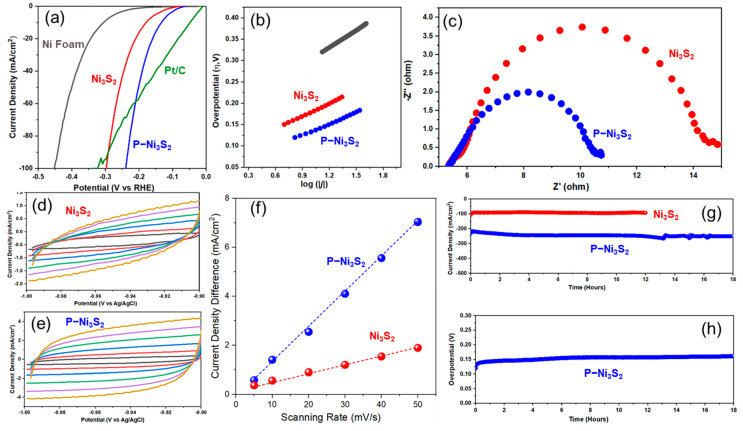
Electrochemical HER performances. (**a**) Polarization curves of Ni foam, Ni_3_S_2_ and P-Ni_3_S_2_, and Pt/C powder, respectively. (**b**) Tafel slopes of Ni foam, Ni_3_S_2_, and P-Ni_3_S_2_ electrodes, derived from the polarization curves in (**a**). (**c**) Impedance spectra for Ni_3_S_2_ and P-Ni_3_S_2_ at −1.3 V vs. Ag/AgCl in 1 M NaOH. (**d**,**e**) Cyclic voltammetry curves (−1~0.9 V vs. Ag/AgCl) for Ni_3_S_2_ and P-Ni_3_S_2_ electrodes at scanning rates of 5, 10, 20, 30, 40 and 50 mV/s, corresponding to CV curves arranged from inner to outer. (**f**) Current density difference at −0.95 V vs. Ag/AgCl versus the scanning rates, derived from (**d**,**e**). (**g**) Long-term stability of Ni_3_S_2_ and P-Ni_3_S_2_ electrodes under a constant applied potential of 1.5 V vs. Ag/AgCl. (**h**) Stability of P-Ni_3_S_2_ electrode under a constant current density of 10 mA/cm^2^.

The electrocatalytic activity of the developed catalysts toward HER under 1 M KOH alkaline electrolyte was evaluated using a typical three-electrode system, in which the catalyst was directly used as the working electrode. Figure 5a shows the polarization curves (linear sweep voltammetry, LSV) of NF, Ni_3_S_2_, P-Ni_3_S_2_, and Pt/C (20 wt.%), respectively. To achieve a current density of 10 mA/cm^2^ for HER, NF requires an overpotential of 306 mV. In contrast, the corresponding HER overpotential for Ni_3_S_2_ is 181 mV, a substantial decrease of 125 mV compared to NF, indicating the significant activity of sulfides toward alkaline HER activity. A further remarkable improvement happens to the P-Ni_3_S_2_ catalyst with an overpotential of only 131 mV, a decrease of 50 mV owing to the phosphorus doping. For comparison, the Pt/C (20 wt.%) was also tested for alkaline HER under identical operation conditions. That demonstrates to be the most efficient catalyst with an overpotential of 48 mV at 10 mA/cm^2^. To gain insight into the high HER activity for the P-Ni_3_S_2_ catalyst, the Tafel slopes were calculated, as shown in Figure 5b. The P-Ni_3_S_2_ catalyst reveals a Tafel slope of 84.6 mV/dec, much smaller than those of NF (120.7 mV/dec) and Ni_3_S_2_ catalyst (103.7 mV/dec), respectively. Fundamentally, hydrogen evolution in alkaline media occurs in two steps. The first step is the Volmer step, where the catalyst accepts one electron and cleaves the adsorbed H_2_O molecule into a hydroxyl ion (OH^−^) and an adsorbed hydrogen atom (H*), i.e., H_2_O + e^−^ + * → H* + OH^−^ (* denotes active site on the electrode surface). In the second step, hydrogen intermediates detach to form H_2_ through either the electrochemical interaction between H* and H_2_O molecule with the assistance of one electron (Heyrovsky step, H_2_O + e^−^ + H* → H_2_ + OH^−^) or directly by chemical recombination of two H* (Tafel step, H* + H* → H_2_) [38,39]. Based on the Tafel slope, the HER pathway could be predicted. When the Volmer step, i.e., water adsorption and dissociation, is the rate-determining step, the theoretical Tafel slope is around 120 mV/dec. If the Heyrovsky step is dominant, a Tafel slope of around 40 mV/dec is expected. When the Tafel slope is as low as 30 mV/dec, the HER predominately occurs through the Tafel step [38]. In this study, the Tafel slopes for all three samples range between 40 and 120 mV/dec. However, the value for the NF electrode is close to 120 mV/dec, indicating that the HER on NF in 1 M KOH is governed by water dissociation on the surface. In contrast, the Ni_3_S_2_ catalyst exhibits a Tafel slope of around 103.7 mV/dev, which suggests much faster reaction kinetics with the water dissociation reaction. Given the smallest Tafel slope (84.6 mV/dec) for the P-Ni_3_S_2_ catalyst, it can be apparently concluded that phosphorus doping in Ni_3_S_2_ has significantly accelerated the alkaline HER kinetics and rate. According to the Tafel equation, *η* = *a* + *b* log *j*, where *b* is the Tafel slope, *j* is the current density, the exchange current density *j*_0_, therefore, could be obtained from the Tafel slope intersection with the abscissa [17,38]. The P-Ni_3_S_2_ catalyst displays an exchange current density *j*_0_ of 0.31 mA/cm^2^, nearly double that of the Ni_3_S_2_ catalyst (0.16 mA/cm^2^) and 7 times higher than that of the NF electrode (4.4 × 10^−2^ mA/cm^2^), respectively. This further highlights the superior HER kinetics on the P-Ni_3_S_2_ electrode. To obtain more precise quantitative kinetic information and understand the origin of HER enhancement in P-Ni_3_S_2_ catalyst, electrochemical impedance spectroscopy (EIS) studies were acquired, as shown in Figure 5c. The EIS experimental data were approximated with a two CPE model equivalent circuit, from which the charge-transfer resistance R_ct_ of the HER could be extracted. The P-Ni_3_S_2_ catalyst demonstrates a smaller charge-transfer resistance of 5.35 Ω for the HER reaction, apparently lower than that of the Ni_3_S_2_ catalyst (9.61 Ω). This implies that phosphorus doping facilitates the charge transfer during the HER process.

To elucidate the intrinsic origin of the HER activity improvement, the electrochemically active surface area was determined by measuring the double-layer capacitance (C_dl_) and calculating the roughness factor R_f_. The CVs were recorded within the potential range from −1.0 to −0.9 V vs. Ag/AgCl, with a scan rate varying from 5 to 50 mV/s. In this potential range, no faradaic reactions occur, and the current arises solely from the charging and discharging of the electrical double layer [17,38]. The nearly rectangular CV shape (Figure 5d,e) for both electrodes indicates that, within this potential range, the electrode works as an electrical double-layer capacitor. The difference in current density at −0.95 V vs. Ag/AgCl was plotted against the scan rate, as shown in Figure 5f, revealing a linear relationship. As a result, the *C*_dl_ was determined to be 34 mF/cm^2^ for Ni_3_S_2_ and 143 mF/cm^2^ for P-Ni_3_S_2_. Consequently, the corresponding surface roughness R_f_ was calculated to be 566 and 2383, respectively. Comparison of the C_dl_ and R_f_ reveals that the electrochemically active surface area of the P-Ni_3_S_2_ electrode increases by 4.2 times relative to the Ni_3_S_2_ electrode. The stability of the electrode catalyst is a key parameter. Figure 5g compares the chronoamperometric curves of Ni_3_S_2_ and P-Ni_3_S_2_ at 1.3 V vs. RHE. Upon application of potential, the Ni_3_S_2_ catalyst quickly reached a current density of ~100 mA/cm^2^ and remained stable for at least 12 h of testing. In contrast, the P-Ni_3_S_2_ started at ~220 mA/cm^2^, slightly increased, and stabilized at 250 mA/cm^2^ after 18 h of testing. The P-Ni_3_S_2_ catalyst was further assessed for 18 h with a constant current density of 10 mA/cm^2^, as shown in the chronopotentiometric curve in Figure 5h, which shows a steady potential input of ~1.13 V vs. RHE. Overall, both sulfide catalysts demonstrated robust stability, observed both under applied potential and current generation.

**Figure 6:**
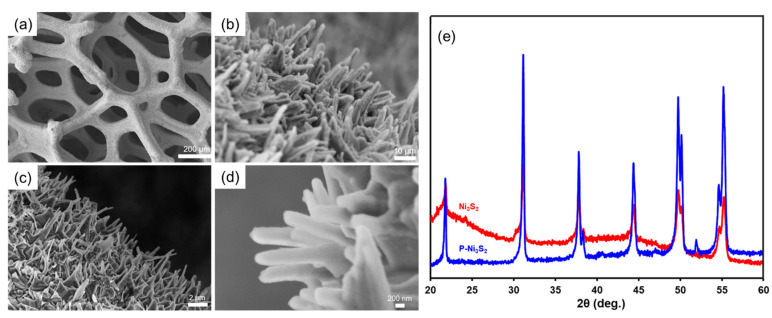
Surface morphology and crystal structure of Ni_3_S_2_ and P-Ni_3_S_2_ electrodes after HER testing. SEM images for Ni_3_S_2_ (**a**,**b**) and P-Ni_3_S_2_ (**c**,**d**) catalysts after HER long-term stability testing with varying magnifications. (**e**) XRD patterns for Ni_3_S_2_ and P-Ni_3_S_2_ catalysts after HER long-term stability testing.

The electrodes were characterized following HER testing. As illustrated in Figure 6, the surface morphology remained intact overall (Figure 6a). Close-up views of both the Ni_3_S_2_ (Figure 6b) and P-Ni_3_S_2_ (Figure 6c,d) catalysts further confirm that the primary nanowire structure was preserved after HER testing. XRD analysis of these tested samples (Figure 6e) revealed that the crystal structure remained consistent with Ni_3_S_2_, with no obvious new phases detected.

### 3.4. Surface Chemistry and Electronic Structure Evolution

**Figure 7:**
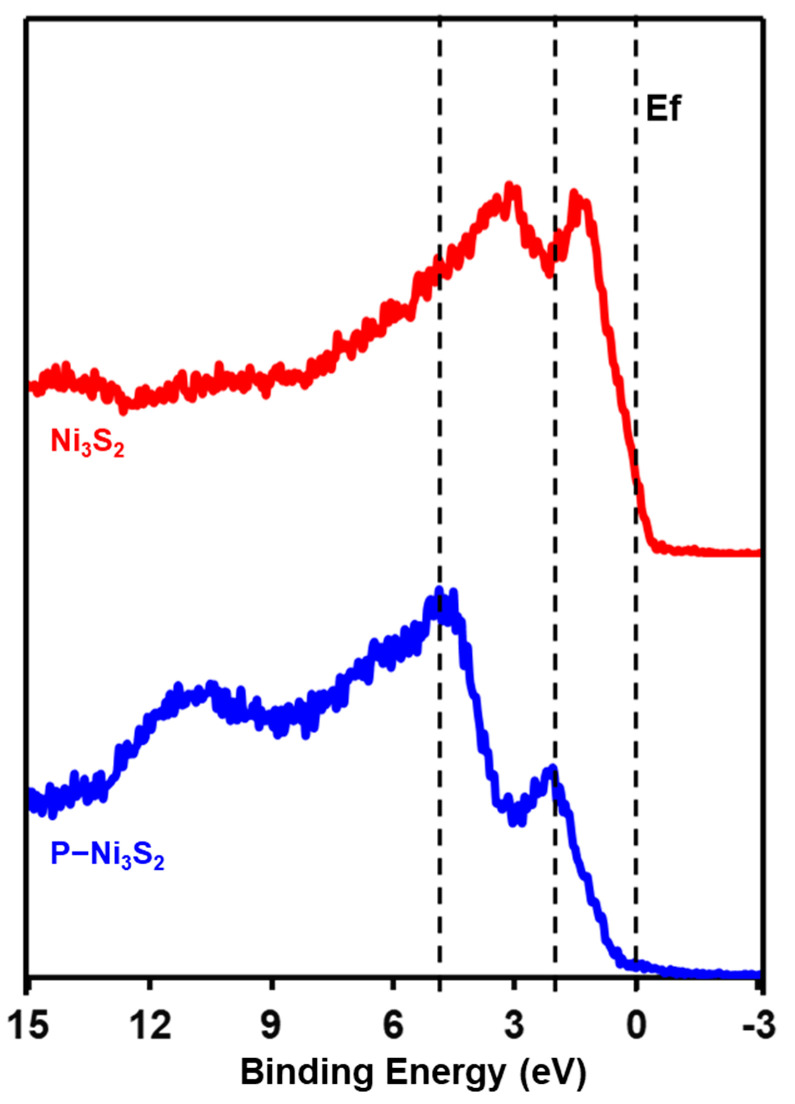
XPS valence band spectra (VBs) of pristine Ni_3_S_2_ and P-Ni_3_S_2_.

The surface electronic density of states (DOS) plays a crucial role in determining charge transfers and energy conversion during surface catalytic reactions [19,33]. According to *d*-band center theory, changes in the *d*-band energy levels relative to the Fermi level influence the formation of anti-bonding states, thereby affecting the adsorption strength of intermediates in electrochemical reactions. Therefore, enhancing the *d*-band center could improve the catalyst’s ability to interplay with intermediates. This is typically achieved by incorporating heteroatoms to modify electronic properties [25,40]. Introducing other metals, such as Co, Mo, or Fe, into the Ni_3_S_2_ structure can shift the *d*-band center, optimizing hydrogen binding energy and boosting HER activity [22,24,25,26,27,28,29]. For instance, Co doping can enhance electronic conductivity and favorably adjust the *d*-band position [25,31]. To shed light on the evolution of the electronic structure, the XPS valence band spectra (VBS) for pristine Ni_3_S_2_ and P-Ni_3_S_2_ were acquired and analyzed, as shown in Figure 7. The primary peak in the valence band spectrum, located at approximately 2.3 eV, is mainly attributed to Ni 3d states, with a minor contribution from S 3p states due to hybridization between Ni 3d and S 3p orbitals. The features at 3.5 eV and 7.5 eV primarily arise from S 3p states [41]. Significant changes in the DOS near the Fermi level were observed in P-Ni_3_S_2_ compared to Ni_3_S_2_, highlighting the effect of phosphorus doping. Specifically, (i) the *d*-band shifted downward, moving further away from the Fermi level, and (ii) the DOS contribution from the *d*-band notably decreased. This trend suggests a potential degradation in the HER performance of P-Ni_3_S_2_, which, however, contradicts the electrochemical performance results. This discrepancy inspires us to further explore the surface chemical states and electronic structure after steady HER testing.

**Figure 8:**
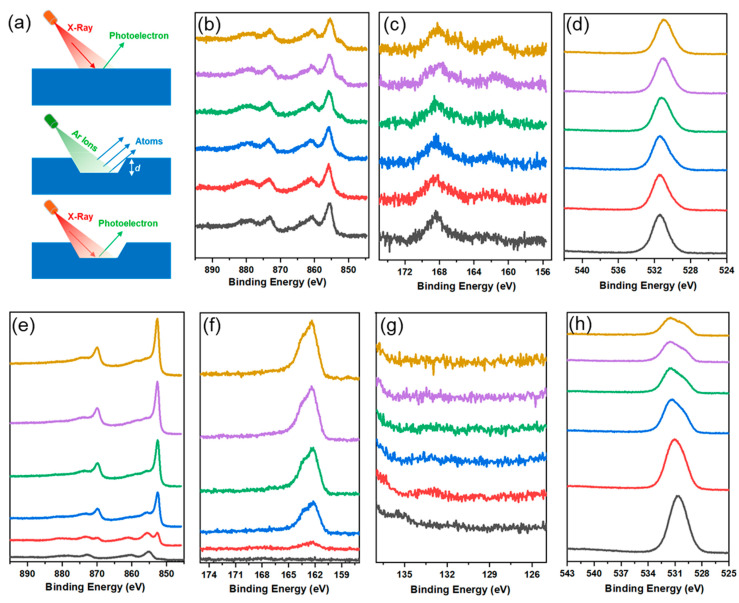
XPS depth profiles of Ni_3_S_2_ and P-Ni_3_S_2_ electrodes after HER long-term stability testing. (**a**) Schematic of XPS depth profiling on catalysts, high-resolution XPS depth profiling spectra for Ni 2p (**b**), S 2p (**c**), and O1s (**d**) of Ni_3_S_2_ catalyst; and Ni 2p (**e**), S 2p (**f**), P 2p (**g**) and O1s (**h**) of P-Ni_3_S_2_ catalyst, respectively. The curves, arranged from bottom to top, represent the surfaces after sputtering cycles ranging from 0 to 5.

Surface restructuring has been observed in phosphide and chalcogenide electrocatalysts during the OER/HER process [3,6,8,9,10,11,12,13]. This phenomenon involves the in situ formation of a new layer of active species initiated on the top of the parent electrode, which plays a crucial role in governing catalytic activity during electrochemical processes. In this study, XPS depth profile characterization was employed for the first time, to the best of our knowledge, to investigate the reconstructed layer in the sulfide electrode. During depth profile acquisition, an ion gun is used to etch the material for a specific duration, after which it is turned off to allow for XPS spectra collection (Figure 8a). Each etching cycle exposes a new surface, and the XPS is used to analyze the compositions of these surfaces. Combining XPS depth profiling with ion beam etching is a powerful technique for studying the chemical composition of functional materials, spanning from the surface to the bulk [42,43]. This approach aims to provide insights into the new active species and enhance our understanding of the origins of HER improvement.

Figure 8 presents the high-resolution XPS depth profiling spectra of the major elements in HER-tested Ni_3_S_2_ and P-Ni_3_S_2_. Notably, both electrodes exhibited distinctly different surface chemistry compared to the pristine samples. In the Ni_3_S_2_ electrode, Ni is primarily in a high valence state, with the major Ni 2p2/3 peak located at 855.7 eV (Figure 8b). Sulfur exhibits an S 2p binding energy of 168.4 eV (Figure 8c), which is assigned to its coordination with oxygen to form S-O bonds and is further confirmed by O 1s spectra in Figure 8d. Meanwhile, the peak for the S-Ni bond, located at around 162 eV, is very weak on the top surface and gradually becomes stronger with increasing sputtering cycles. Correspondingly, after three sputtering cycles, a subtle Ni 2p2/3 peak appears at approximately 852.1 eV, indicating the presence of the Ni-Ni bonds alongside the emergence of the S-Ni bond. For the P-Ni_3_S_2_ electrode, sulfur (Figure 8f) and phosphorus (Figure 8g) completely disappeared from the very top layer, while nickel predominantly existed in a high valence state, with the main Ni 2p2/3 peak located at 855.7 eV (Figure 8e). After one sputtering cycle, the S 2p signal corresponding to sulfides appeared, centered around 162 eV, while the Ni 2p2/3 peak associated with Ni-Ni bonds sharply increased and became dominant. This suggests that the layer beneath the outermost surface layer retained the Ni_3_S_2_ crystal structure in the P-Ni_3_S_2_ electrode, as demonstrated by the XPS spectra of the pristine samples (Figure 4). Notably, the phosphorus signal did not appear even after five sputtering cycles (Figure 8g). In comparison, it is concluded that the reconstructed layer is significantly thicker in the Ni_3_S_2_ electrode than in the P-Ni_3_S_2_ electrode. This is consistent with the XRD patterns in Figure 6e, where the Ni_3_S_2_ electrode showed lowered diffraction intensity. Since no S and P are detected on the top surface, the presence of phosphorus likely promotes the formation of a very thin Ni-O layer, as shown in the Ni 2p (Figure 8e) and O1s (Figure 8h) spectra, which inhibits further restructuring of the inner structure and is responsible for HER activity.

**Figure 9:**
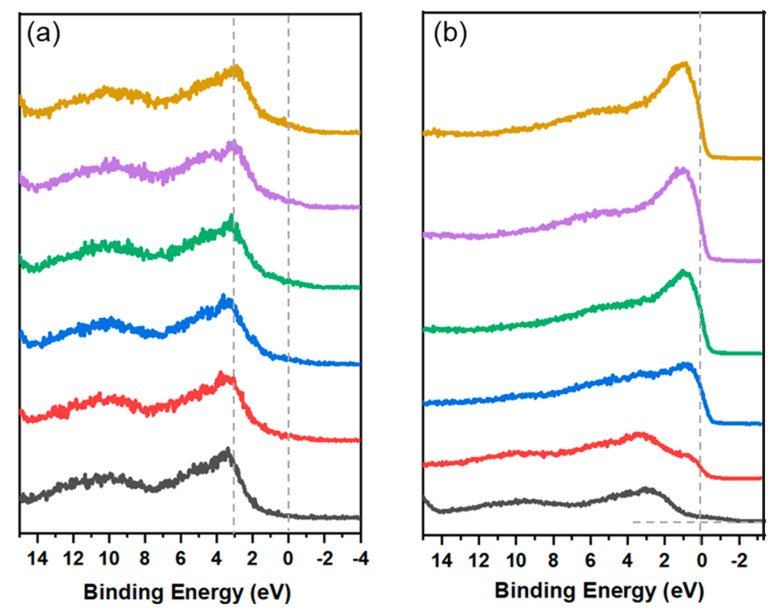
XPS depth profiles of valence band spectra for Ni_3_S_2_ (**a**) and P-Ni_3_S_2_ (**b**) catalysts after HER long-term stability testing. The curves, arranged from bottom to top, represent the surfaces after sputtering cycles ranging from 0 to 5.

The XPS depth profiles of valence band spectra were also recorded after each sputtering cycle, as shown in Figure 9. Due to surface restructuring, the VB spectra exhibited distinct differences compared to the respective pristine electrodes for both Ni_3_S_2_ and P-Ni_3_S_2_. These spectral changes indicate subtle modifications in the electronic structure [41]. The newly reconstructed layer shifts the *d*-band in the Ni_3_S_2_ electrode away from the Fermi level to 3.5 eV, resulting in a decrease in the density of states near the Fermi level (Figure 9a), compared to the pristine Ni_3_S_2_ electrode. Additionally, the VB of the Ni_3_S_2_ electrode remains relatively unchanged with increasing sputtering cycles. However, it is worth noting that after three sputtering cycles, a subtle shift of the major peak towards E_f_ occurred (Figure 9a). This observation aligns well with the emergence of Ni-Ni and S-Ni bonds shown in Figure 8b,c. In contrast, the outer Ni-O layer in the P-Ni_3_S_2_ electrode shows a peak at 3 eV, with a tail crossing over the Fermi level (Figure 9b), which contributes to enhanced electrical conductivity, as illustrated in the EIS spectra (Figure 5c). As revealed in Figure 9, after one sputtering cycle, the intensity of the *d*-band crossing the Fermi level significantly increased, enriching the DOS near the Fermi level. As a consequence, this optimization facilitates better binding energy of reactants and intermediates with active sites for alkaline HER, while the heterointerfaces enhance overall charge transfer within the electrode.

### 3.5. Discussion

Surface restructuring has been confirmed on both sulfide electrodes, and it is important to highlight that the chemistry of transition-metal sulfides is highly dynamic and closely dependent on the electronic structure of the parent catalysts. In this study, phosphorus doping in Ni_3_S_2_ resulted in a thin reconstructed layer composed solely of Ni and O, contrasting with the pure Ni_3_S_2_-reconstructed active layer, which includes Ni, O, and S. This compositional difference, clearly illustrated in XPS data (Figure 8), significantly influences catalytic activity. The reconstructed Ni-O layer exhibits superior performance in the P-Ni_3_S_2_ electrode. Alkaline HER begins with the water dissociation process, which involves overcoming an energy barrier for H_2_O rotation on the catalyst surface to achieve optimal *p*-*d* bonding overlap [40]. Since the Fermi level is primarily influenced by the 3*d* orbitals of Ni, the upshifted *d*-band and increased density of states near the Fermi level in the reconstructed Ni-O layer enhance its affinity for hydroxyl (OH) species, promoting water dissociation, as indicated by the smaller Tafel slope in Figure 5b. The hydrogen (H^+^) generated from water dissociation will diffuse to either sulfur or oxygen sites in the Ni_3_S_2_-reconstructed layer, but it is more likely to adsorb on oxygen sites in the P-Ni_3_S_2_-reconstructed Ni-O layer. The low-coordinated oxygen sites in the Ni-O layer, formed during surface restructuring, possess strong redox capabilities for H adsorption and desorption [9,10], which contribute to the superior HER activity in the P-Ni_3_S_2_ electrode. Our work highlighted the crucial role of the parent catalyst in surface restructuring, emphasizing that a thorough understanding of this process is essential for designing non-precious metal sulfides to enhance catalytic activity.

## 4. Conclusions

In conclusion, phosphorus-doped Ni_3_S_2_ (P-Ni_3_S_2_) has been synthesized and demonstrated superior alkaline HER activity for hydrogen production. While the crystal structure and morphology were largely retained, the XPS depth profiling revealed that in situ surface restructuring occurred on electrodes during the HER process. A thick restructuring layer containing Ni, O, and S formed on the pure Ni_3_S_2_ electrode, whereas a very thin Ni-O layer was created on top of the P-Ni_3_S_2_ electrode. This in situ-formed Ni-O restructuring layer is responsible for the enhanced HER activity, featuring an increased electrochemical surface area, upshifted *d*-band, and enriched density of states near the Fermi level, which strengthen the adsorption of OH and expedite water dissociation. Furthermore, beneath the thin top Ni-O layer in the P-Ni_3_S_2_ electrode, the crystal structure of Ni_3_S_2_ was preserved, with a strong contribution from Ni 3*d* orbitals to the density of states near the Fermi level. This preservation benefits overall electrical conductivity and charge transfer by forming a heterostructure. This study highlights that a deep comprehension of surface restructuring is vital for further enhancing and predicting the catalytic activity of transition-metal sulfide electrocatalysts and calls for further investigation in this area.

## Data Availability

The original contributions presented in this study are included in the article. Further inquiries can be directed to the corresponding authors.
